# The Effect of Prebiotics and Probiotics on Levels of Depression, Anxiety, and Cognitive Function: A Meta‐Analysis of Randomized Clinical Trials

**DOI:** 10.1002/brb3.70401

**Published:** 2025-03-04

**Authors:** Atefeh Zandifar, Rahim Badrfam, Mahdi Mohammaditabar, Bita Kargar, Saba Goodarzi, Amirhossein Hajialigol, Shera Ketabforoush, Afshin Heidari, Hanie Fathi, Arman Shafiee, Hadi Pourjafar

**Affiliations:** ^1^ Dietary Supplements and Probiotic Research Center Alborz University of Medical Sciences Karaj Iran; ^2^ Clinical Research Development Unit of Imam Hossein Medical Education Center Alborz University of Medical Sciences Karaj Iran; ^3^ Social Determinants of Health Research Center Alborz University of Medical Sciences Karaj Iran; ^4^ Department of Psychosomatic Medicine Shariati Hospital, Alborz University of Medical Sciences Karaj Alborz Iran; ^5^ Non‐communicable Diseases Research Center Alborz University of Medical Sciences Karaj Alborz Iran; ^6^ Community Mental Health Center Alborz University of Medical Sciences Karaj Alborz Iran; ^7^ Student Research Committee, School of Medicine Alborz University of Medical Sciences Karaj Iran; ^8^ Alborz Office of Universal Scientific Education and Research Network (USERN) Alborz University of Medical Sciences Karaj Iran; ^9^ Tehran Medical Sciences Islamic Azad University Tehran Iran; ^10^ Student Research Committee Tehran Medical Sciences Islamic Azad University Tehran Iran; ^11^ School of Medicine Isfahan University of Medical Sciences Isfahan Iran

**Keywords:** anxiety, cognitive function, depression, gut microflora, mental disorders, prebiotics, probiotics

## Abstract

**Introduction:**

Recent studies have emphasized the relationship between mental health and the human intestine microbiota. In this study, we evaluate the effect of consuming Biotics, on levels of depression, anxiety, and cognitive function.

**Methods:**

This meta‐analysis adhered to the Preferred Reporting Items for Systematic Reviews and Meta‐Analyses (PRISMA) standards. We searched MEDLINE (PubMed), Cochrane Library, Scopus, Web of Science, and ClinicalTrials.gov. All full‐text articles and major reviews were manually searched for additional studies.

**Results:**

The initial analysis was based on the concept that consuming Biotics causes changes in anxiety, measured using various instruments. This analysis showed that consuming Biotics significantly reduced anxiety in our study participants (SMD = 0.2894, *Z* = 2.46, *P* = 0.0139, *I*^2 = 92.4%). The meta‐analysis included 4295 samples (2194 in the experimental group and 2101 in the control group). In terms of depression, the analysis showed that consuming Biotics significantly reduced depression in our study participants (SMD = 0.2942, *Z* = 2.13, *P* = 0.0335, *I*^2 = 91.7%). The meta‐analysis included 3179 samples (1603 in the experimental group and 1576 in the control group). Regarding cognitive function, the analysis showed that consuming Biotics significantly improved cognitive function in our study participants (SMD = 0.4819, *Z* = 3.00, *P* = 0.0027, *I*^2 = 77.9%). The meta‐analysis included 915 samples (470 in the experimental group and 445 in the control group).

**Conclusions:**

Our results indicate that most recent studies support the effectiveness of probiotics in reducing symptoms of anxiety, depression, and cognitive issues despite some discrepancies in the findings. People with mild symptoms may experience greater benefits from taking probiotics.

**Trial Registration:**

PROSPERO registration ID: CRD42024589507.

## Graphical abstract

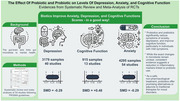

**Abbreviations**
CFS: chronic fatigue syndrome
GAD: generalized anxiety disorder
HPA: hypothalamic–pituitary–adrenal
IL‐10: Interleukin‐10
MDD: major depressive disorder
PHES: Psychometric Hepatic Encephalopathy Score
TNF: tumor necrosis factor

## 1 Introduction

The prevalence of anxiety and depressive disorders is increasing worldwide and it leads to important morbidity and mortality (Marazziti et al. 2014). Causes of depression may include neurological, environmental, genetic, personality, and inflammatory factors (Beck and Bredemeier 2016; Disner et al. 2011). Another cause of the patient's anxiety and depression may be cognitive impairment (McCollum and Karlawish 2020).

Probiotics and prebiotics are two essential food components that influence physiological processes through the gastrointestinal tract. Probiotics are defined as live microorganisms that, when consumed, yield beneficial effects in preventing and treating particular pathological conditions. These microorganisms are believed to function through a process known as colonization resistance, where the native anaerobic flora restricts the growth of potentially pathogenic (mainly aerobic) microorganisms in the digestive system. Other mechanisms, such as the provision of enzymes or the modulation of enzyme activity within the gastrointestinal tract, may also contribute to the physiological benefits attributed to probiotics (Chow 2002). On the other hand, prebiotics are nondigestible food ingredients that enhance host health by selectively stimulating the growth and/or activity of a limited number of bacteria in the colon (Rauch et al. 2022).

Through regular aging, there are changes in the central nervous system, causing age‐associated mood disorders and mental impairment, which are the main health problems among the elderly. Due to the increasing age of most developed countries' populations, neurological diseases' importance has become clearer (Mattson and Arumugam 2018) and there exists a pressing requirement to manage the upward trend in age‐standardized incidence rates of neurological disorders across Western Europe, East Asia, and Southern Latin America, notably in the nations of Italy, China, and Ecuador (Huang et al. 2023).

Existing treatments, psychotherapy, and pharmacotherapy have limits, for instance, patient variable effectiveness, adverse effects, and delayed therapeutic onset (Al‐Harbi 2012). This has spurred interest in alternative or adjunctive therapies that are both effective and have minimal side effects (Ansari et al. 2020).

Over 38 trillion bacterial cells live inside the human gastrointestinal tract, called the intestinal microbiota (Sender et al. 2016). The gut microbiota is an important part of the gut–brain axis, which plays an important role in the immune and neuroendocrine, enteric, and autonomic nervous systems (Skonieczna‐Żydecka et al. 2018; Dinan and Cryan 2017).

In recent decades, there has been significant research on changes in the intestinal microbiota. One key area of study has been the consumption of biotics, which can be categorized as probiotics—live microorganisms that when taken in adequate amounts, provide health benefits to the host, prebiotics—nonviable food components that contribute to the host's health by affecting the microbiota, and Synbiotic—combined food components containing both probiotics and prebiotics (Pandey et al. 2015). Recent research has emphasized the relationship between neurodegenerative diseases and the human intestine microbiota (Quigley 2017; Chandra et al. 2020; Jemimah et al. 2023). Also, several studies show that microbiota change can cause anxiety and mood disorders (Liśkiewicz et al. 2021; Mason et al. 2020; Madan et al. 2020). Meanwhile, prebiotic consumption and diet changes decrease the risk of mood disorders and improve mild‐to‐moderate depression (Ansari et al. 2020; Dash et al. 2015; Zhang et al. 2023). Another study suggests the inflammation caused by the reaction of the gut bacteria with lipopolysaccharide (a portion of Gram‐negative bacteria's outer membrane), might play a role in the pathogenesis of human depression (Cani et al. 2007; Maes et al. 2012). Given these connections, the therapeutic potential of prebiotics and probiotics in improving cognitive function alongside mood disorders is a compelling area of investigation (Ansari et al. 2023).

The growing interest in this field has led to multiple systematic reviews and meta‐analyses addressing the relevant topic (Liśkiewicz et al. 2021; Mason et al. 2020; Madan et al. 2020). Unlike these previous works, our study focused on a more uniform cohort by excluding participants with comorbid depression and healthy controls. Furthermore, we employed a more extensive range of interventions, including prebiotics and probiotics, and utilized diverse outcome measures to assess their effectiveness. Importantly, our research incorporated several recent studies that were not part of earlier reviews. The primary aim of this study is to investigate whether management strategies for gut microbiota can significantly influence the levels of depression, anxiety, and cognitive function. Specifically, we seek to evaluate the effectiveness of prebiotics and probiotics on levels of depression, anxiety, and cognitive function symptoms, while also exploring factors that may affect their efficacy and summarizing the changes in gut microbiota as reflected by various indices, along with shifts in biochemical indicators related to the levels of depression, anxiety, and cognitive function. Despite the promising indications from individual studies, the literature on this topic is characterized by variability in study designs, sample sizes, and outcomes, leading to inconclusive results. A meta‐analysis of randomized clinical trials (RCTs) is warranted to address these inconsistencies and provide a more definitive evaluation. This meta‐analysis aims to systematically review and synthesize the existing evidence on the effects of prebiotics and probiotics supplementation on levels of depression, anxiety, and cognitive function.

## 2 Methods

### 2.1 Study Design and Data Sources

This meta‐analysis adhered to the Preferred Reporting Items for Systematic Reviews and Meta‐Analyses (PRISMA) standards (1). From their inception to May 5, 2024, we conducted an extensive literature search in databases including MEDLINE (PubMed), Cochrane Library, Scopus, Web of Science, and ClinicalTrials.gov. The search strategy included phrases such as “prebiotic* OR probiotic*” AND “cognit*[tiab] OR sleep [tiab] OR anxiety*[tiab]” AND “trial*.” The results were exported to the EndNote X9 program for further screening (Supplement 1).

The search strategy for each database is given below:

PubMed:

(prebiotic* OR probiotic* OR postbiotic* OR “dietary fiber” OR synbiotic*) AND (cognit*[tiab] OR sleep[tiab] OR anxiet*[tiab]) AND (trial[tiab] OR trial[pt] OR trial*)

Scopus:

(prebiotic* OR probiotic* OR postbiotic* OR “dietary fiber” OR synbiotic*) AND (cognit* OR sleep OR anxiet*) AND (trial*)

Web of Science:

(prebiotic* OR probiotic* OR postbiotic* OR “dietary fiber” OR synbiotic*) AND (cognit* OR sleep OR anxiet*) AND (trial*)

### 2.2 Eligibility Criteria

**FIGURE 1:**
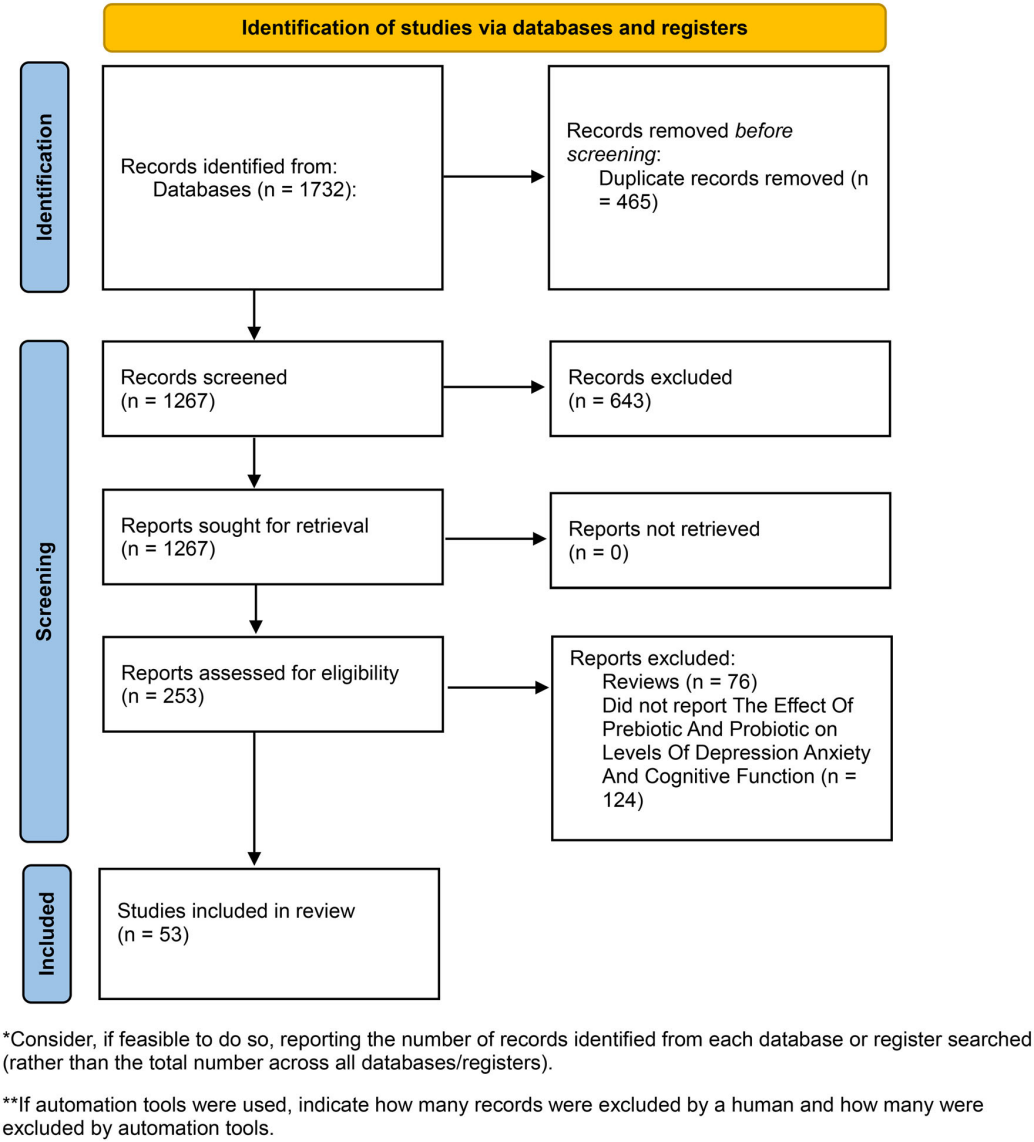
PRISMA 2020 flow diagram for new systematic reviews which included searches of databases and registers only.

Studies that met the following criteria were included: (1) randomized controlled trials (RCTs) examining the effects of prebiotics, probiotics, or synbiotics on cognitive performance, anxiety, and depression. (2) Research presenting trials with validated outcome measures for anxiety (e.g., Beck Anxiety Inventory), depression (e.g., Hamilton Depression Rating Scale), and cognitive function (e.g., Mini‐Mental State Examination). (3) Published in peer‐reviewed journals with accessible English full text) (Figure 1).

### 2.3 Study Selection

Two independent reviewers evaluated the titles and abstracts of selected papers. The full texts of potentially eligible studies were retrieved and assessed for possible inclusion. Disagreements were resolved through discussion or consultation with a third reviewer.

### 2.4 Data Extraction

Three reviewers independently extracted data using a standardized form. Extracted data included authors, country, year of publication, sample size, intervention, assessment tool, outcomes, descriptions of control and intervention arms, and total number of participants in each trial. Mean and standard deviations of qualified studies were used for meta‐analysis.

### 2.5 Quality Assessment

The quality of included studies was evaluated using the Cochrane Risk of Bias 2 (ROB‐2) Tool, considering incomplete outcome data, selective reporting, blinding of participants and personnel, blinding of allocation concealment, random sequence generation, and other biases. All studies were rated as “Low,” “Some concerns,” or “High” based on the checklist. Conflicts were resolved through consensus or by consulting a third reviewer.

### 2.6 Statistical Analysis

Meta‐analysis and statistical analysis were performed using R (San Francisco, USA). We used standardized mean differences as the summary statistic for continuous data by attaining the experiment and placebo groups' mean (SD) and sample size (*n*). All data were analyzed at a single point at the end of the trial. The effect of using different types of biotics (e.g., Probiotic, Prebiotic, or Synbiotic) or utilizing different measuring tools (indexes) was assessed by subgrouping our meta‐analysis. Between‐study heterogeneity was investigated using the I2 test since an I2 of around 25, 50, and 75% is considered low, moderate, and high levels of heterogeneity, respectively. The random‐effects model was supposed to be calculated. Publication bias was assessed using a funnel plot and Egger's test. Egger's linear regression test was used to evaluate asymmetry, and *P* < 0.05 was set as the significance level.

## 3 Results

### 3.1 Effect of Consuming Biotics on Anxiety

**FIGURE 2:**
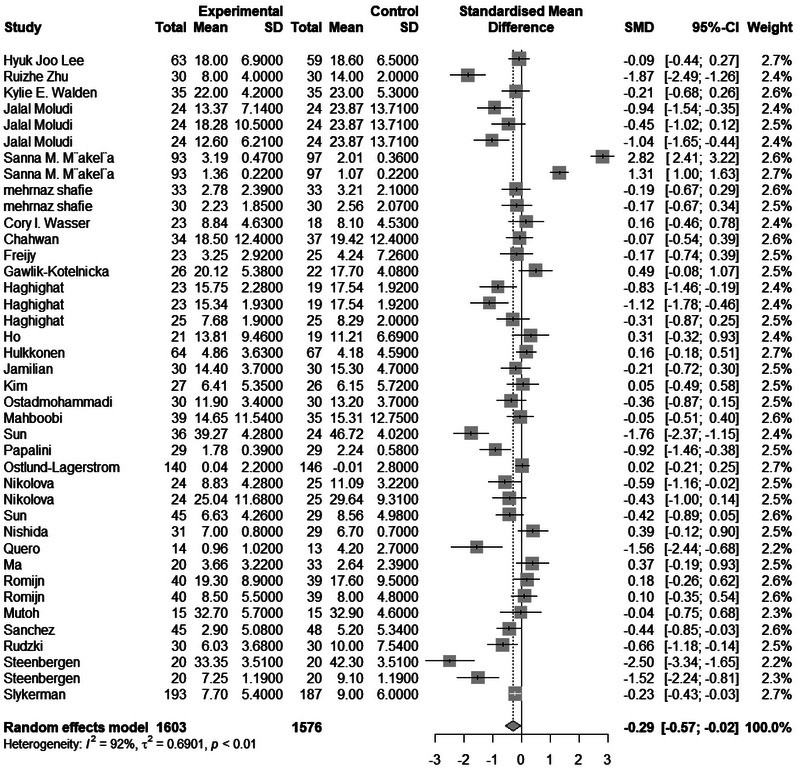
Forest plot and effect of consuming Biotics on Anxiety.

**FIGURE 3:**
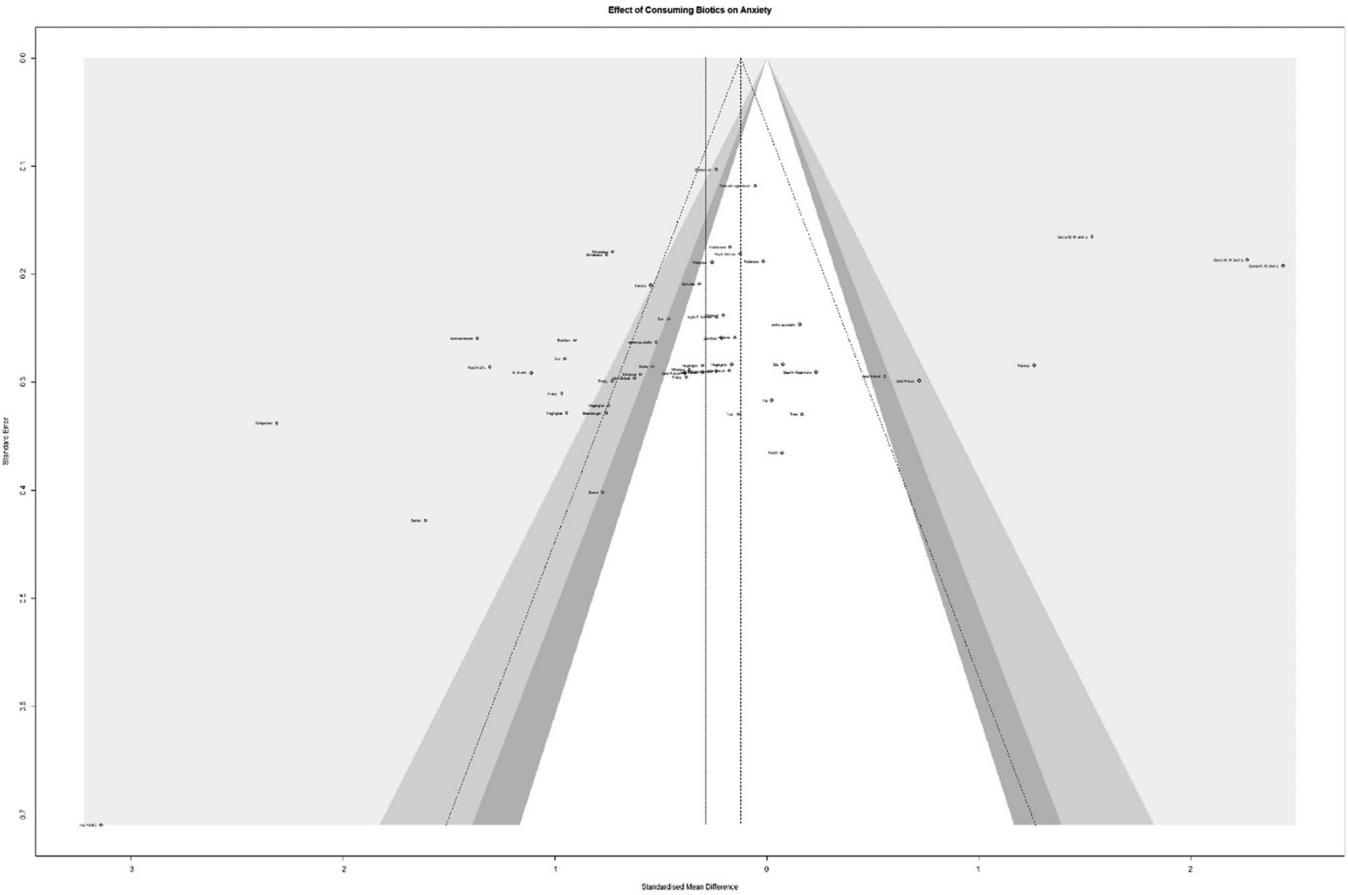
Funnel plot and effect of consuming Biotics on Anxiety.

The meta‐analysis included 4295 samples (experiment = 2194, control = 2101) from 54 studies. The initial analysis was based on the concept that consuming any Biotics causes any changes in anxiety based on any measuring instruments. This analysis showed that consuming Biotics has significantly reduced the Anxiety of our observance (SMD = 0.2894, *Z* = 2.46, *P* = 0.0139, *I*^2 = 92.4%) (Figure 2). The data were further analyzed by subgroups based on the type of Biotics (e.g., Probiotic, Prebiotic, or Synbiotic) and measuring instruments (e.g., BAI, HAMA, STAI, etc.). There were differences between types of Biotics (*P* < 0.0001), except samples that used both probiotics and prebiotics. All subgroups reduced the Anxiety. Also, there were differences between measuring instruments (*P* < 0.0001); most measuring instruments measured the reduction of Anxiety, and 5 out of 18 different measuring instruments showed opposite results. After conducting a leave‐one‐out analysis to detect outliers, it was found that all the pooled estimates after removing one survey at a time were still within the 95% confidence interval of the overall estimate (Supplement 1). However, the funnel plot and Egger's test results indicated the presence of funnel plot asymmetry (95% CI [−6.19 to −0.43]/*p* value = 0.0285) (Figure 3).

### 3.2 Effect of Consuming Biotics on Depression

**FIGURE 4:**
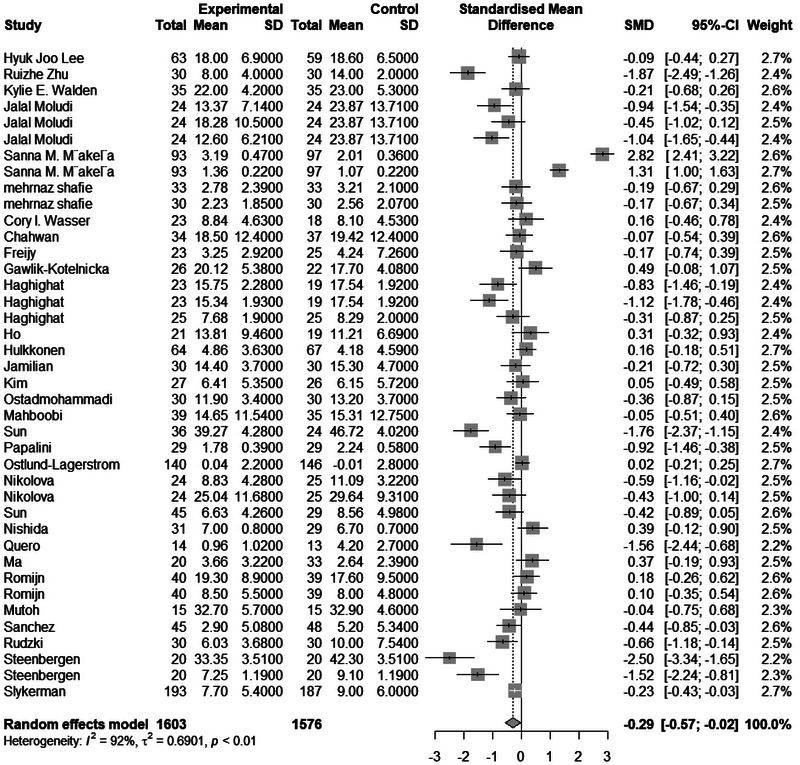
Forest plot and effect of consuming Biotics on Depression.

**FIGURE 5:**
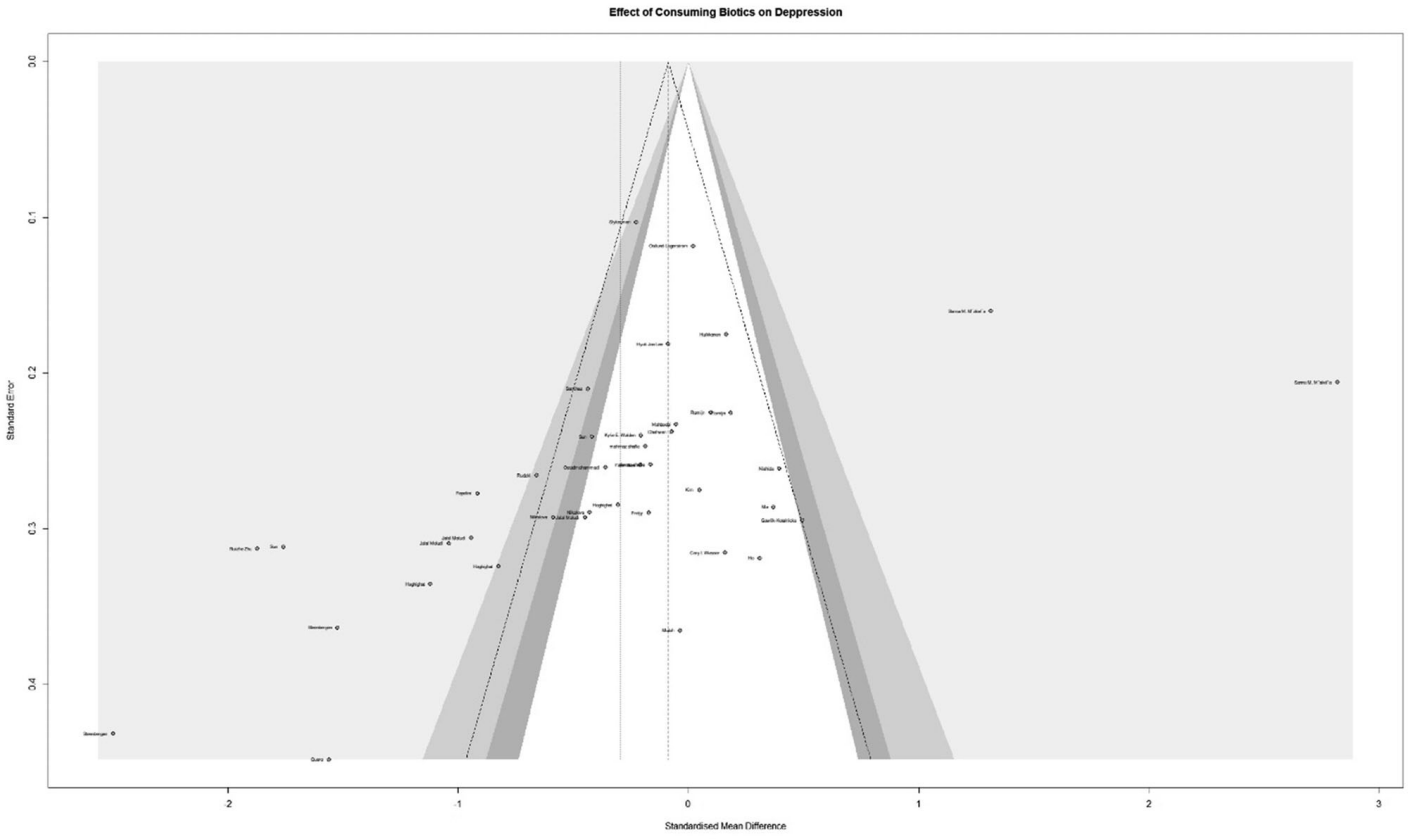
Funnel plot and effect of consuming Biotics on Depression.

The meta‐analysis included 3179 samples (experiment = 1603, control = 1576) from 40 studies. The initial analysis was based on the concept that consuming any Biotics causes any changes in Depression based on any measuring instruments. This analysis showed that consuming Biotics has significantly reduced the Depression of our observance (SMD = 0.2942, *Z* = 2.13, *P* = 0.0335, *I*^2 = 91.7%) (Figure 4). The data were further analyzed by subgroups based on the type of Biotics and measuring instruments (e.g., BDI, HAMD, DASS, etc.). There were differences between types of Biotics (*P* < 0.0017), but all subgroups showed a reduction of the Depression although it was not significant. Also, there were differences between measuring instruments (*P* < 0.0001); 7 out of 15 different measuring instruments showed opposite results but most samples used the BDI instrument, which measured the reduction of Depression. After conducting a leave‐one‐out analysis to detect outliers, it was found that all the pooled estimates after removing one survey at a time were still within the 95% confidence interval of the overall estimate (Supplement 1). However, the funnel plot and Egger's test results indicated the presence of funnel plot asymmetry (95% CI [−6.85 to −0.81]/*p* value = 0.0176) (Figure 5).

### 3.3 Effect of Consuming Biotics on Cognitive Function

**FIGURE 6:**
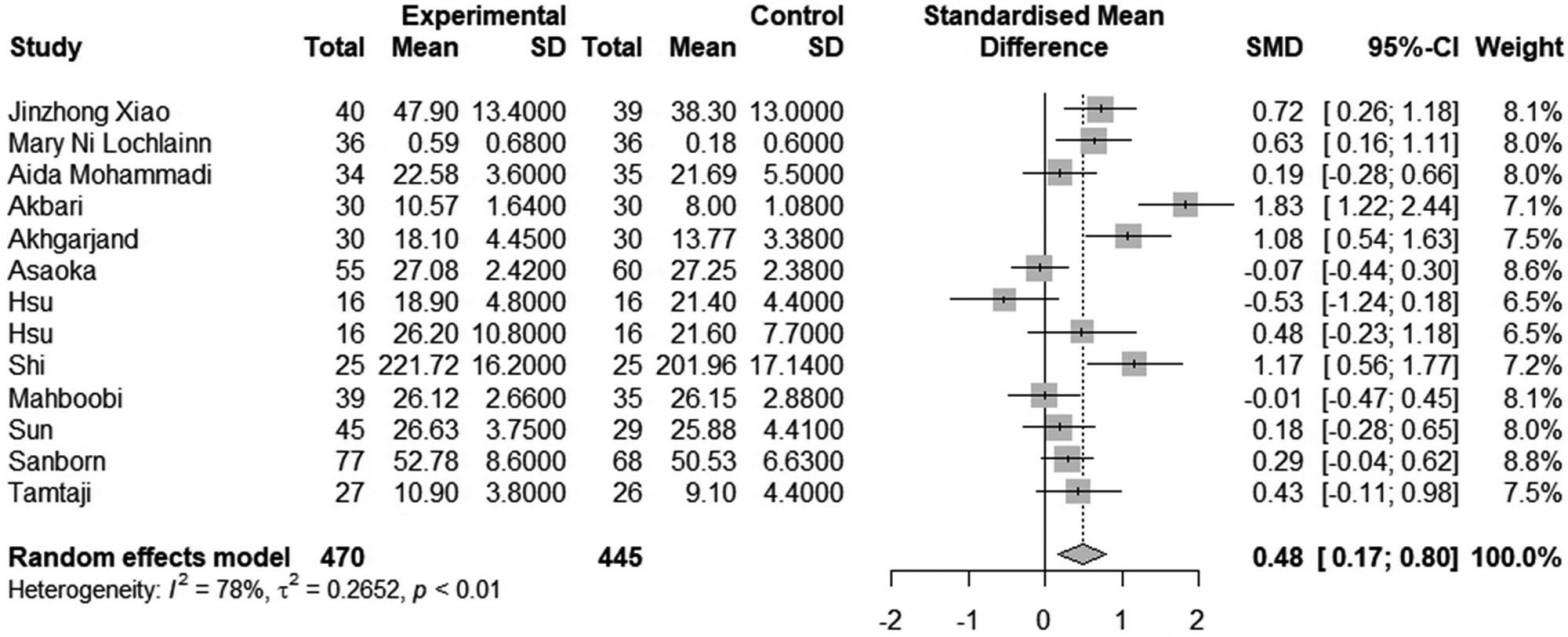
Forest plot and effect of consuming Biotics on Cognitive Function.

**FIGURE 7:**
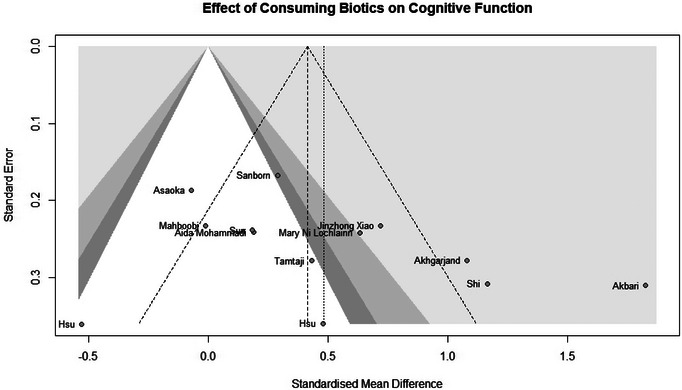
Funnel plot and effect of consuming Biotics on Cognitive Function.

The meta‐analysis included 915 samples (experiment = 470, control = 445) from 13 studies. The initial analysis was based on the concept that consuming any Biotics causes any changes in Cognitive Function based on any measuring instruments. This analysis showed that consuming Biotics has significantly improved the Cognitive Function of our observance (SMD = 0.4819, *Z* = 3.00, *P* = 0.0027, *I*^2 = 77.9%) (Figure 6). The data were further analyzed by subgroups based on the type of Biotics and measuring instruments (e.g., RBANS, MoCA, MMSE, etc.). There were no significant differences between types of Biotics (*P* = 0.4060), except the observation that used probiotics and vitamin D. All subgroups significantly improved Cognitive Function. Also, there were no significant differences between measuring instruments (*P* = 0.0702). Only two out of six different measuring instruments showed improvement in Cognitive Function. After conducting a leave‐one‐out analysis to detect outliers, it was found that all the pooled estimates after removing one survey at a time were still within the 95% confidence interval of the overall estimate (Supplement 1). However, the funnel plot and Egger's test results did not indicate the presence of funnel plot asymmetry (95% CI [−1.64–8.54]/*p* value = 0.2115) (Figure 7) (Supplements 2–5).

## 4 Discussions

Our meta‐analysis revealed that the impact of prebiotics and probiotics on depression, anxiety, and cognitive function levels was significantly more effective than that of a placebo. Subgroup and meta‐regression analyses were conducted to investigate the factors associated with therapeutic efficacy, and the findings are further elaborated upon in this study. The results demonstrated that consuming Biotics has significantly improved depression, anxiety, and cognitive function levels, as they remained significant across different assessment scales. Moreover, consuming Biotics improved mild‐to‐moderate depression symptoms, serving as potential alternatives for those with this severity range, as antidepressant drugs were found to be more effective in severe depression. Additionally, Chahwan et al. reported that probiotic intervention led to lower cognitive vulnerability to depression in patients with mild‐to‐moderate severity (Chahwan et al. 2019).

The majority of studies comparing probiotics, prebiotics, and synbiotics have utilized probiotics as the primary intervention. The results of subgroup analysis unveiled substantial pooled effects of interventions incorporating probiotics (i.e., probiotics and synbiotics) in improving depressive symptoms. Nonetheless, only one study focusing on synbiotics demonstrated significant antidepressant effects. Furthermore, the outcomes from three trials on prebiotics did not show any significant distinction compared to the placebo group (Ghorbani et al. 2018; Heidarzadeh‐Rad et al. 2020; Kazemi et al. 2019; Kazemi et al. 2019; Tarutani et al. 2022). Liu et al. (year) conducted a meta‐analysis on prebiotics and probiotics for depression, aligning with our findings. They found no significant difference between prebiotics and control conditions in reducing depressive symptom scores, while probiotics showed significant antidepressant effects (Liu et al. 2019). The current lack of comprehensive studies on prebiotics and synbiotics' effects means it is too early to draw firm conclusions about their effectiveness in treating depressive symptoms. As a result, it is strongly advised that further clinical trials be conducted to delve into the potential therapeutic properties of prebiotics and synbiotics in the future. In terms of probiotics, common strains such as Bifidobacterium (e.g., B. longum, B. bifidum, and B. breve) and Lactobacillus (e.g., L. casei and L. acidophilus) are typically found in probiotic capsules, acting as beneficial microbial flora. L. casei is commonly used to manage gastrointestinal diseases, with limited evidence supporting its efficacy in treating depression. Since its discovery in 1990, L. acidophilus has been recognized for its ability to alleviate lactose intolerance, enhance the immune function of the host, and impede the progression of cardiovascular disease (Gao et al. 2022). Within the Bifidobacterium genus, B. breve has emerged as a promising candidate for addressing psychiatric disorders by eliciting antidepressant effects through mechanisms like the modulation of the hyperactive axis of hypothalamic–pituitary–adrenal (Tian et al. 2020). Similarly, B. longum has been shown to diminish limbic reactivity, thereby reducing responses to negative emotional stimuli in the brain (Pinto‐Sanchez et al. 2017). The ongoing identification and utilization of novel probiotic strains offer a diverse range of options for selecting appropriate probiotics for the treatment of depression. Furthermore, prebiotics, probiotics, and synbiotics have been used as supplementary treatments in numerous studies. However, there is currently insufficient evidence to support their substitution for antidepressant medications as a primary treatment. Notably, the duration of treatment with prebiotics, probiotics, and synbiotics did not impact their efficacy based on subgroup analyses. Studies with interventions lasting over 8 weeks yielded nonsignificant results, likely due to the incorporation of prebiotics in those trials.

A significant combined effect for probiotics and anxiety emerged in the current review, contrasting with the outcomes of two recent studies (Liu et al. 2018; Reis et al. 2018). The current meta‐analysis is stronger for several reasons. It not only addresses methodological limitations observed in previous analyses but also includes a larger number of studies, enhancing the statistical power to detect a significant small effect, as demonstrated in this case. Moreover, one prior meta‐analysis of anxiety by Liu et al. (Liu et al. 2018) showed significant heterogeneity, whereas the other by Reis et al. (2018) did not exhibit significant heterogeneity after the removal of an outlier, unlike the current review. This discrepancy between meta‐analyses may be partly due to the broader definition of anxiety adopted in the current review, including outcomes like visceral sensitivity.

A total of 13 studies have indicated a positive impact of probiotics on cognition (Asaoka et al. 2022; Xiao et al. 2020; Mohammadi et al. 2024; Ni Lochlainn et al. 2024; Akbari et al. 2016; Akhgarjand et al. 2022; Hsu et al. 2023; Shi et al. 2022; Mahboobi et al. 2022; Sanborn et al. 2020; Tamtaji et al. 2019). This evidence points towards the potential therapeutic benefits of probiotics for individuals experiencing cognitive impairments due to various conditions. Studies have shown improvements in cognition among young and middle‐aged adults with HIV‐1, MDD, Fibromyalgia, and CFS (Ceccarelli et al. 2017; Rudzki et al. 2019; Roman et al. 2018). It is worth noting that most of these effects were observed in individual studies, some of which were open‐label and not randomized control trials. However, reports of enhanced cognition were more consistent in cirrhosis patients, with three out of four randomized control trials demonstrating improvements in PHES composite score and related subtests (Lunia et al. 2014; Román et al. 2019). Despite methodological limitations, the existing evidence in these clinical populations is encouraging and warrants further investigation.

The results of the subgroup and meta‐regression analysis indicated that gender significantly influenced the efficacy of the intervention. Several studies have highlighted the impact of gender on the composition of gastrointestinal microbial, whether in healthy individuals or patients with depression. It was found that healthy females had a higher abundance of the Bacteroides genus compared to males. Conversely, males exhibited a higher abundance of Veillonella and Escherichia genera in their gut microbiota than females (Haro et al. 2016; Singh and Manning 2016). When examining microbiota patterns in depressed patients, it was observed that drug‐free females experiencing their first depressive episode had increased levels of Actinobacteria, while males had decreased levels of Bacteroides (Chen et al. 2018).

Estrogen may have a role in influencing the difference in microbial composition between sexes. Research has demonstrated that removing the ovaries in mice can lead to an imbalance in gut microbes (Sinha et al. 2019; Org et al. 2016). Considering the variations in gut microbiota between the sexes, the biological sex of depressive patients might also affect how they respond to treatments targeting gut microbiota, such as probiotics, prebiotics, and synbiotics (Vemuri et al. 2019). A study by Karunasena et al. involved feeding mice a probiotic (L. animalis). The findings showed that Staphylococcus and Roseburia genera were consistently more abundant in females than in males, suggesting that the host's response to probiotics was influenced by sex. (Karunasena et al. 2014).

Following the intervention, there were distinct microbial alterations based on sex, alongside varying immune responses observed between males and females (Vemuri et al. 2019). Mu et al. discovered that the administration of Lactobacillus had an anti‐inflammatory effect by decreasing IL‐6 levels and enhancing IL‐10 production in the gastrointestinal tract of female and castrated male mice, whereas intact males did not exhibit the same response (Mu et al. 2017). The differences in gut microbiota and immune system activity based on sex may elucidate the sex‐specific amelioration of depressive symptoms following treatment with probiotics, prebiotics, and synbiotics. Given that the majority of research studies have predominantly included female subjects and have paid limited attention to sex‐related efficacy disparities, future investigations should delve into the interplay among sex, depression, and gut microbiota.

The potential antidepressant effects of probiotics, prebiotics, and synbiotics may be explained by various mechanisms associated with our secondary outcomes. The primary and most direct function of probiotics, prebiotics, or synbiotics was their interaction with the gut microbiota and its ecosystem. Abildgaard et al. in an animal trial revealed a difference in the internal microbiota composition between individuals who responded positively and those who did not respond to probiotics concerning depressive‐like behavior. The study found that the fecal abundance of certain genera, particularly the genus of Lactobacillus, was higher in responders compared to nonresponders (Abildgaard et al. 2019). In essence, probiotics produce antidepressant effects by modifying the internal microbiota. Moreover, the utilization of antibiotics can increase the vulnerability to depression by impacting the gut microbiota. Ido Lurie et al. employed a comprehensive population‐based medical record database in the United Kingdom to execute three nested case‐control studies, demonstrating a link between antibiotic use and a heightened risk of depression. Furthermore, recurrent exposure to antibiotics was found to amplify this risk (Lurie et al. 2015). Another research endeavor utilized antibiotic mixtures to induce depression in a mouse model, resulting in alterations in depression‐related biomarkers. The composition of intestinal microbiota in mice with antibiotic‐induced depression also underwent notable modifications, including an increase in Bacteroides and Klebsiella (Fan et al. 2022).

Probiotics, however, have been shown to effectively restore gut microbiota dysregulation. Our quantitative analysis of the change in α diversity following the intervention did not show significant results compared to the placebo, while qualitative analysis of β diversity yielded inconsistent findings. The insufficient sample sizes in the analyses and the specific number of microbial species in each study may explain these outcomes. Previous research has indicated that patients with MDD had lower levels of the Bifidobacterium genus compared to controls (Aizawa et al. 2016; Liu et al. 2016). However, our analysis revealed an increase in the Bifidobacterium genus after the intervention, along with improvements in depressive symptoms. Further research is necessary to elucidate the specific association between gut microbiota and depression treatment, including prebiotics, probiotics, and synbiotics. The second mechanism, immune modulation, is also relevant. Patients with MDD were found to have elevated proinflammatory cytokines and acute phase proteins such as IL‐6, TNF, and C‐reactive protein in their blood (Beurel et al. 2020). Several studies have highlighted the anti‐inflammatory properties of probiotics and prebiotics (Wall et al. 2010; Desbonnet et al. 2010). Probiotics can enhance levels of anti‐inflammatory cytokines like TNF, while prebiotics have been shown to diminish type 2 T helper responses (Sanders et al. 2019). It is worth noting that the effects of different probiotics or prebiotics can vary, with some exhibiting pro‐inflammatory effects and others displaying more anti‐inflammatory properties. This variability contributes to the lack of significant findings when comparing inflammatory markers between intervention and placebo groups. Moreover, the limited number of studies included in the analyses is a crucial factor (Klaenhammer et al. 2012). These microbiota management agents may also influence the brain through various pathways, including the vagus nerve, HPA axis, microbial metabolites, and the neurotransmitter serotonin (Margolis et al. 2021). Therefore, future investigations should delve into these mechanisms to expand our current knowledge of microbiota management agents.

Our review identified limited evidence on the effects of probiotics on the diversity of gut microbiota and the abundance of select genera. Previous research has pointed to associations between major depressive disorder (MDD), generalized anxiety disorder (GAD), and an increase in Proteobacteria and Enterobacteriaceae abundance, supporting our observation that probiotic consumption was linked to a reduction in Proteobacteria levels (Jiang et al. 2015; Jiang et al. 2018). Additionally, our analysis suggested that probiotics could elevate Lactobacillus abundance (Schaub et al. 2022; Lee et al. 2021), with certain strains like L. casei, L. rhamnosus, and multistrain products containing L. plantarum potentially influencing psychiatric disorders and stress‐related behaviors (Zhu et al. 2023). However, numerous studies failed to detect significant alterations in microbiota composition, potentially due to participants' unchanged dietary and lifestyle habits during short‐term probiotic intake. Findings on alpha and beta diversity changes were scarce and contradictory. In essence, it was challenging to discern concrete evidence of specific microbiome changes following probiotic usage based on our review.

## 5 Limitations

The review conducted has several limitations. First, it is plausible that the studies analyzed in this review predominantly focused on individuals with mild‐to‐moderate anxiety or depression, as those with severe mental illnesses may have been less inclined or able to participate in clinical trials. Furthermore, certain studies within our review targeted specific patient populations (such as cancer, irritable bowel syndrome, and insomnia), potentially restricting the applicability of the findings to a broader patient base. Most of the studies were carried out in a single center and were based on relatively small sample sizes. Moreover, there was a wide variation in the types of probiotic supplements utilized and the duration of treatment, factors that could have impacted the outcomes. Additionally, it was challenging to draw a definitive conclusion on whether probiotics served as the primary or adjunct intervention due to inconsistencies in the psychopharmacological/psychological treatments administered alongside probiotics, with a significant number of studies failing to control for or report this information. Some studies included participants with mental health conditions like MDD or GAD, while others focused on individuals with subclinical depressive or anxiety symptoms. The population under study exhibited substantial heterogeneity, with some studies lacking adequate information on patient treatment and potential confounding variables such as the duration of mental illness. Lastly, several studies relied solely or partially on self‐reported data, while others incorporated clinical assessments.

## 6 Conclusions

The current comprehensive analysis examines the latest data from clinical trials conducted over the past 10 years regarding the impact of probiotics on the management of anxiety, depression, and cognitive function. Our results suggest that despite some discrepancies in the outcomes, the majority of recent studies seem to endorse the efficacy of probiotics in mitigating symptoms of anxiety, depression, and cognitive function. Individuals with mild symptoms may experience greater benefits from taking probiotics. Furthermore, while a definitive conclusion on probiotic‐induced changes in the microbiome remains elusive, a significant body of evidence supports a decrease in inflammatory markers linked to probiotic consumption.

Probiotics offer a nonpharmacological approach to treating mood disorders, potentially broadening treatment options for both psychiatric and nonpsychiatric patients. This method is considered safe and well‐tolerated, which could help reduce the social stigma associated with psychotropic medications. Despite advancements in understanding their mechanisms and effectiveness, there is still a knowledge gap concerning the optimal use of probiotics. Further research is required to determine if probiotics can be used together with antidepressants or as a standalone treatment for depression. Additionally, more investigation is needed to assess their effectiveness across different medication combinations. Ongoing studies should focus on elucidating the precise mechanisms of probiotic action, determining appropriate dosages and treatment durations, and assessing potential variations in outcomes based on the severity of anxiety and depression. Since the specific bacteria responsible for alleviating depressive symptoms have not been definitively identified, future investigations should explore various probiotic combinations, as well as the individual effects of symbiotic and prebiotic treatments. Additionally, due to the complexity of analyzing diverse probiotic‐related factors, such as interventions, populations, and outcome measures, researchers are encouraged to adhere to expert consensus guidelines when designing studies in this area (McFarland et al. 2023).

## Author Contributions

**Atefeh Zandifar**: methodology, project administration, supervision, visualization, validation, writing–review and editing. **Rahim Badrfam**: methodology, validation, visualization, writing–review and editing, supervision. **Mahdi Mohammaditabar**: conceptualization, data curation, investigation, methodology, project administration, resources, software, writing–original draft. **Bita Kargar**: conceptualization, investigation, writing–original draft, methodology, software, project administration, data curation, resources. **Saba Goodarzi**: conceptualization, investigation, writing–original draft, methodology, software, project administration, data curation, resources. **Amirhossein Hajialigol**: formal analysis, supervision, validation, visualization, writing–review and editing. **Shera Ketabforoush**: conceptualization, investigation, methodology, software, data curation, resources, project administration, writing–original draft. **Afshin Heidari**: conceptualization, data curation, investigation, methodology, project administration, resources, writing–original draft, software. **Hanie Fathi**: writing–original draft, conceptualization, investigation, methodology, project administration, software, data curation, resources. **Arman Shafiee**: formal analysis, supervision, visualization, validation, writing–review and editing. **Hadi Pourjafar**: supervision, validation, visualization, writing–review and editing.

## Conflicts of Interest

The authors declare no conflicts of interest.

### Peer Review

The peer review history for this article is available at https://publons.com/publon/10.1002/brb3.70401.

## Supporting information



Supporting Information

Supporting Information

Supporting Information

Supporting Information

Supporting Information

## Data Availability

The data that support the findings of this study are available in the supplementary material of this article.
